# Supplementary material for: IL36 is a critical upstream amplifier of neutrophilic lung inflammation in mice
Source: Commun Biol. 2021 Feb 8;4:172. doi: 10.1038/s42003-021-01703-3 (PMC7870940; doi:10.1038/s42003-021-01703-3)
Supplement: Supplementary file 2 — Supplementary Information [file 42003_2021_1703_MOESM2_ESM.pdf]

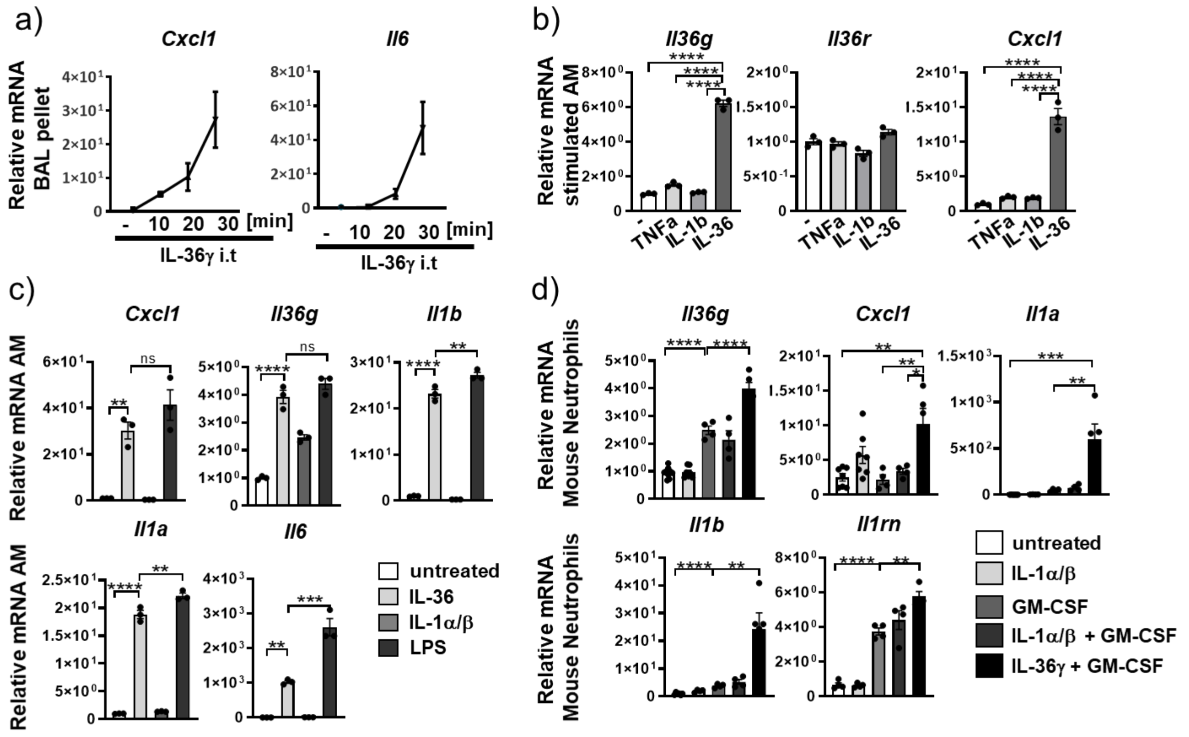

Supplementary Fig 1 IL-36γ as an upstream inflammatory driver in mouse neutrophils and alveolar macrophages. a Cxcl1 and Il6 mRNA expression of the BAL pellet from untreated (n=6) and IL-36γ exposed mice (intratracheal instillation) after 10, 20 and 30 min (n=7).**b** *Il36g, Il36r and Cxcl1* mRNA expression in naïve mouse alveolar macrophages (pooled n=15 mice) and stimulated *in vitro* with either no cytokines (-), TNF-a, IL-1β or IL-36αβγ. **c** *Cxcl1, Il36g, Il1b, Il1a,* and *IL6* mRNA expression in naïve mouse alveolar macrophages (pooled n=15 mice) and stimulated *in vitro* with either no cytokines (-), IL-36αβγ, or IL-1α/IL-1β ; or LPS (**d**) *Il36g, Cxcl1, Il1b, Il1a,* and *il1rn* mRNA expression in mouse bone marrow derived neutrophils (from n=4 mice) *in vitro* stimulated with either no cytokines (-), IL-1α/IL-1β ; GM-CSF, IL-1α/IL-1β+GM-CSF or IL-36γ+GM-CSF. *P ≤ 0.05, **P ≤ 0.01, ***P ≤ 0.001, ****P ≤ 0.0001 vs all other groups by one-way ANOVA and Tukey’s correction.

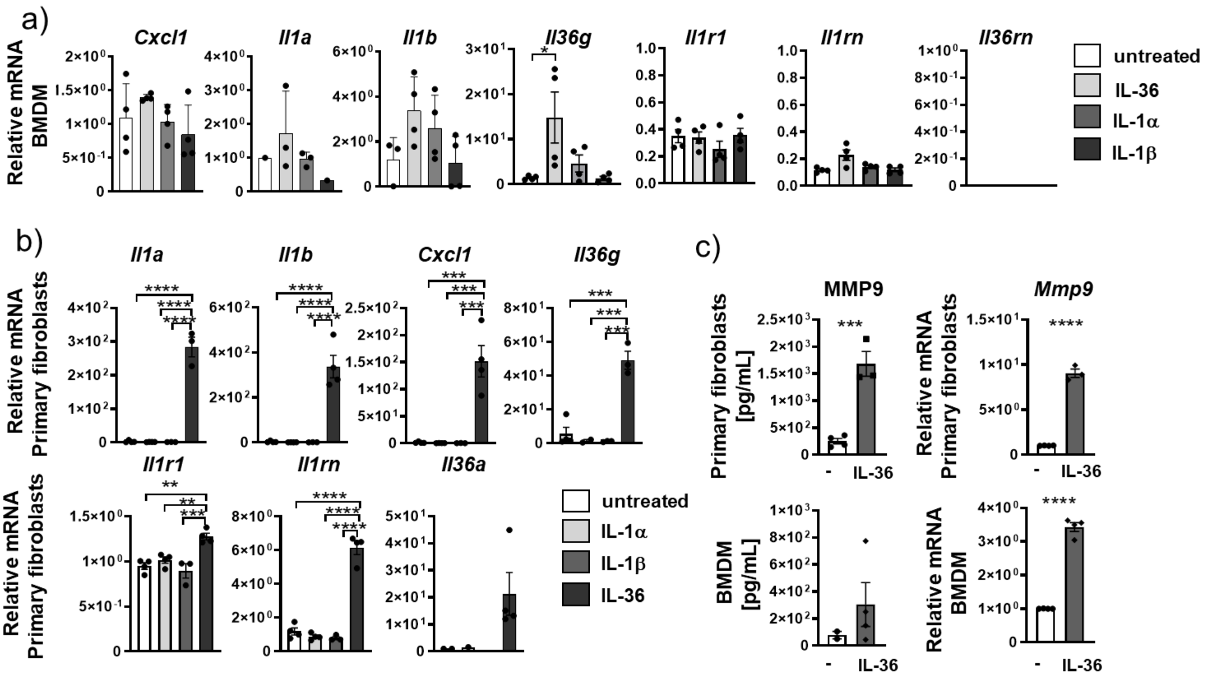

Supplementary Fig 2 IL-36γ as an upstream amplifier in mouse macrophages and fibroblasts. a Relative mRNA amounts in naïve mouse bone marrow derived macrophages (BMDM) (n=4) of *Il36g, Cxcl1, Il1a, Il1b, Il1r1, Il1rn* and *Il36rn* after no stimulation (-) and after stimulation with IL-36αβγ, IL1α, IL1β. **b** Relative mRNA amounts of *Il1a, Il1b, Cxcl1,IL-36g Il1r1, Il1rN,* and *IL-36a* in primary mouse lung fibroblasts (n=4) after stimulation with IL-36γ, IL1α, or IL1β relative to untreated **c** Protein concentrations of MMP-9 in supernatant and relative mRNA amounts of *Mmp9* in primary mouse lung fibroblasts (n=4) and BMDMs (n=4) after IL-36αβγ stimulation relative to no stimulation (-). All data are depicted as mean ± SEM from biological replicates. (**a,b**) *P ≤ 0.05, **P ≤ 0.01, ***P ≤ 0.001, ****P ≤ 0.0001 vs all other groups by one-way ANOVA and Tukey's correction. (**c**) *P ≤ 0.05, **P ≤ 0.01, ***P ≤ 0.001, ****P ≤ 0.0001 vs untreated by *t* test.

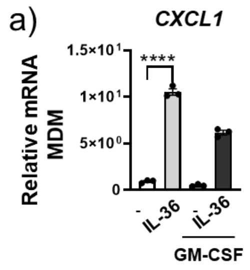

Supplementary Fig 3 IL-36γ as an upstream amplifier in human macrophages and fibroblasts. a Relative mRNA amounts in human monocyte derived macrophages (MDM) (depicted are mean values ± SME of technical triplicates from one representative of four experiments) of *CXCL1* after no stimulation (-), and after stimulation with IL-36αβγ, GMCSF or the combination of IL-36αβγ and GMCSF. ***P ≤ 0.001 vs all other groups by one-way ANOVA and Tukey's correction.

a) Percent reduction BALF vs lung homogenate protein concentrations from Il36r$^{-/-}$

Cytokine	% reduction BALF	% reduction homogenate
IL-1α	62	44
CXCL1	69	47
IL6	54	48
Tnf-a	66	24

b)

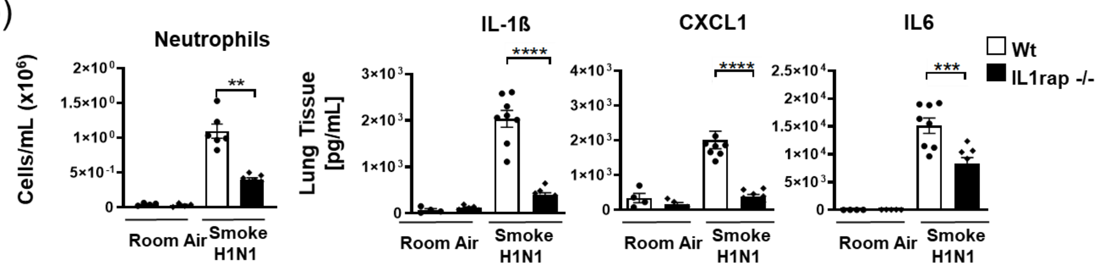

c)

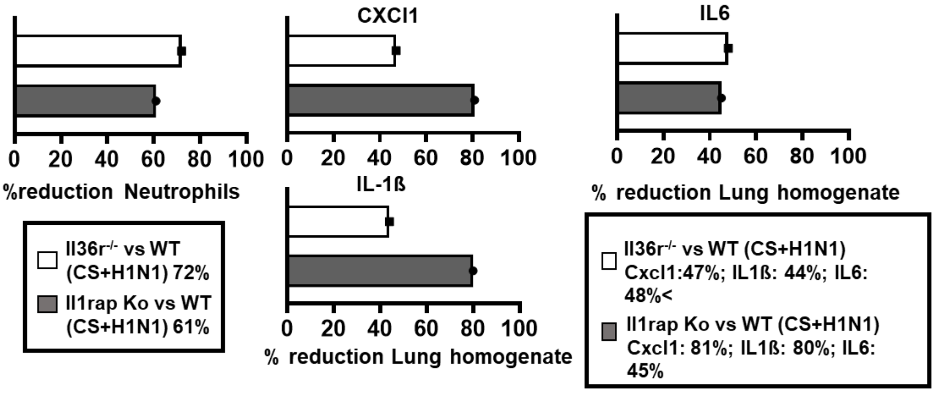

Supplementary Fig 4 IL-36γ is critical in neutrophilic lung inflammation a Percent reduction of IL1α, CXCL1, IL6 and TNF protein concentrations in BALF and lung homogenate of *IL-36r*$^{-/-}$ mice after CS+H1N1 exposure (comparing data in Fig. 5f,g). **b** Neutrophil numbers in BALF and IL1ß, CXCL1 and IL6 protein concentrations in lung homogenate from room air exposed (RA, n=8) and CS+H1N1 (n=8) exposed WT and *Il1rap*$^{-/-}$ mice. **c** Percent reduction of neutrophil numbers in BALF and CXCL1, IL1β and IL6 protein concentrations in lung homogenate from *IL-36r*$^{-/-}$ and *Il1rap*$^{-/-}$ mice after CS+H1N1 exposure (comparing data from Fig. 5g with Suppl. Fig. 4a). Depicted are mean values ± SEM of biological replicates. (**b**) $^{**}P \leq 0.01$, $^{***}P \leq 0.001$, $^{****}P \leq 0.0001$ vs all other groups by one-way ANOVA and Tukey's correction.

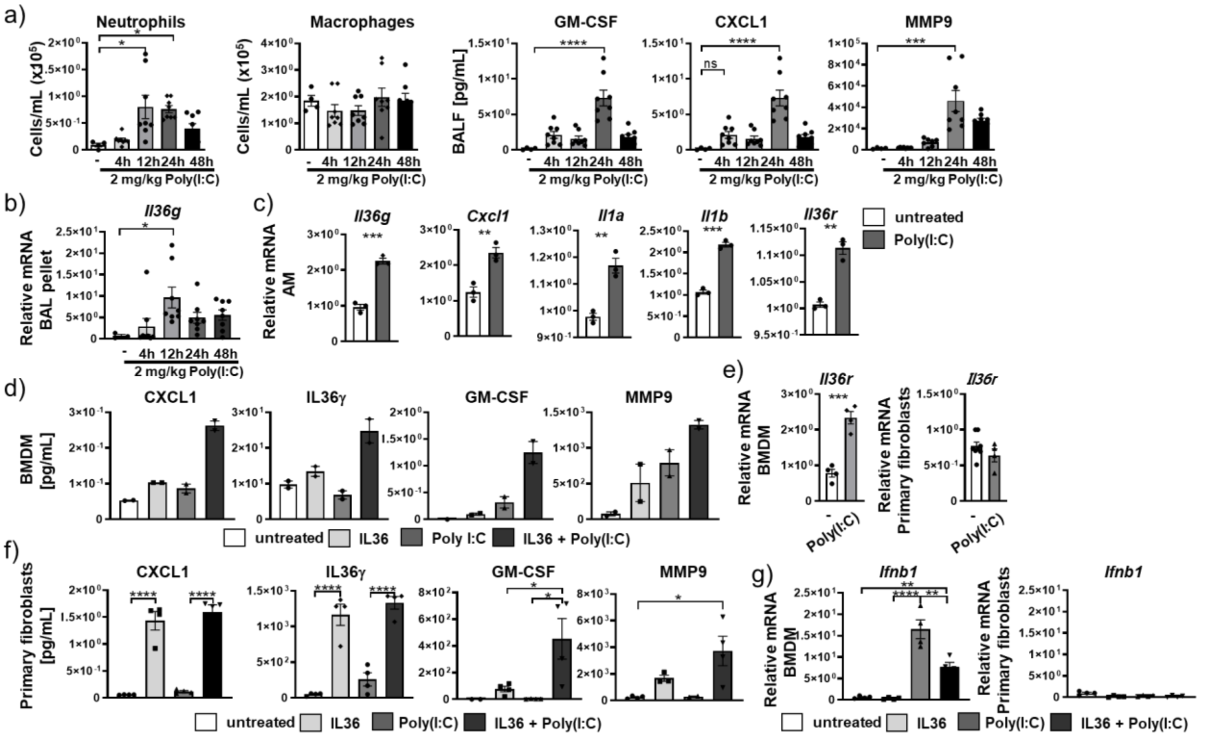

Supplementary Fig 5 IL-36γ cooperates with poly(I:C) on macrophages and fibroblasts a-b Neutrophils and macrophage numbers in BAL and GMCSF, CXCL1 and MMP9 protein concentrations in BALF and relative *Il36g* mRNA amounts in the BAL cell pellet of untreated (n=4) and poly(I:C) exposed mice (n=8) for the indicated amounts of time. **c** Relative mRNA amounts of *Il36g, Cxcl1, Il1a, Il1b* and *IL-36r* in alveolar macrophages stimulated with poly(I:C) *in vitro* (AMs were pooled from 4 naïve). **d;f** CXCL, IL-36γ, GM-CSF and MMP9 protein concentrations in supernatants from BMDM (n=2) and primary mouse lung fibroblasts (n=4) either unstimulated (-) or stimulated with IL-36αβγ, poly(I:C) or the combination of IL-36αβγ and poly(I:C). **e** Relative mRNA amounts of *Il36r* in BMD-M (n=4) and primary mouse lung fibroblasts (n=4) as well as after poly(I:C) stimulation. **g** Relative mRNA amounts of *Infb1* in BMDMs (n=4) and primary mouse lung fibroblasts (n=4) and BMDMs (n=4) after IL-36αβγ, poly(I:C) or the combination of IL-36αβγ and poly(I:C) relative to no stimulation (-). (Depicted are mean values ± SEM of biological replicates). (**a,b,d,f,g**) *P ≤ 0.05, **P ≤ 0.01, ***P ≤ 0.001, ****P ≤ 0.0001 vs all other groups by one-way ANOVA and Tukey's correction. (**c,e**) *P ≤ 0.05, **P ≤ 0.01, ***P ≤ 0.001, ****P ≤ 0.0001 vs untreated by *t* test.

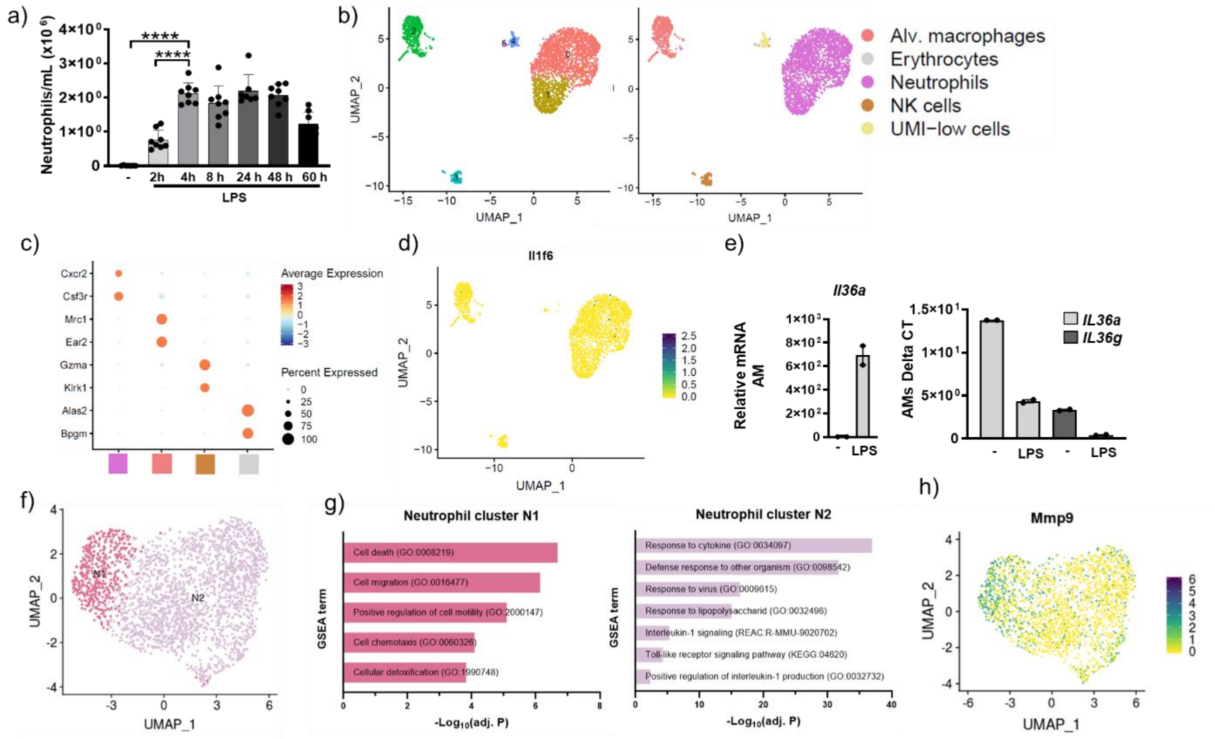

Supplementary Fig 6 Neutrophils are a source of IL-36γ in acute lung injury. a Neutrophil numbers in BAL samples from untreated and 2, 4, 6, 8, 12, 18, 24, 36, 48 and 60 hours post LPS exposure (n=8). **b** UMAP representation of the cell clusters (left) and cell populations (right) identified in BAL of mice 4 h after LPS stimulation. **c** Expression of the marker genes used to annotate the cell types. Data are shown per cell type using the color code used in (a). Scaled average expression levels are indicated by the dot color and dot size indicates the fraction of cells that express the respective marker gene. **d** Visualization of *Il36a* (*Il1f6*) expression levels per single cell. Normalized expression values are indicated by the color code. **e** *IL-36a, IL-36b* and *IL-36g* mRNA expression of alveolar macrophages unstimulated (-), or exposed to LPS for 4 h (Depicted are mean values ± SEM of biological replicates). **f** UMAP representing the subclusters (N1 and N2) identified within the neutrophils from (b). **g** Gene set enrichment analysis (GSEA) of the neutrophil subclusters N1 and N2. Selected pathways are shown per subcluster. **h** Visualization of *Mmp9* expression in neutrophils. Normalized expression levels per single cell are indicated by the color code.
