# Supplementary material for: IL36 is a critical upstream amplifier of neutrophilic lung inflammation in mice
Source: Commun Biol. 2021 Feb 8;4:172. doi: 10.1038/s42003-021-01703-3 (PMC7870940; doi:10.1038/s42003-021-01703-3)
Supplement: Supplementary file 3 — Description of Additional Supplementary Files [file 42003_2021_1703_MOESM3_ESM.pdf]

Description of additional supplementary files

File name: Supplementary Data 1

Description: Source data for the main and supplementary figures
