# Supplementary material for: IL36 is a critical upstream amplifier of neutrophilic lung inflammation in mice
Source: Commun Biol. 2021 Feb 8;4:172. doi: 10.1038/s42003-021-01703-3 (PMC7870940; doi:10.1038/s42003-021-01703-3)
Supplement: Supplementary file 5 — Reporting Summary [file 42003_2021_1703_MOESM5_ESM.pdf]

nature research

Corresponding author(s): Karim El Kasmi

Last updated by author(s): Dec 17, 2020

Reporting Summary

Nature Research wishes to improve the reproducibility of the work that we publish. This form provides structure for consistency and transparency in reporting. For further information on Nature Research policies, see our Editorial Policies and the Editorial Policy Checklist.

Statistics

For all statistical analyses, confirm that the following items are present in the figure legend, table legend, main text, or Methods section.

n/a	Confirmed	
☐	☒	The exact sample size (*n*) for each experimental group/condition, given as a discrete number and unit of measurement
☐	☒	A statement on whether measurements were taken from distinct samples or whether the same sample was measured repeatedly
☐	☒	The statistical test(s) used AND whether they are one- or two-sided<br>*Only common tests should be described solely by name; describe more complex techniques in the Methods section.*
☒	☐	A description of all covariates tested
☐	☒	A description of any assumptions or corrections, such as tests of normality and adjustment for multiple comparisons
☐	☒	A full description of the statistical parameters including central tendency (e.g. means) or other basic estimates (e.g. regression coefficient) AND variation (e.g. standard deviation) or associated estimates of uncertainty (e.g. confidence intervals)
☒	☐	For null hypothesis testing, the test statistic (e.g. *F*, *t*, *r*) with confidence intervals, effect sizes, degrees of freedom and *P* value noted<br>*Give P values as exact values whenever suitable.*
☒	☐	For Bayesian analysis, information on the choice of priors and Markov chain Monte Carlo settings
☒	☐	For hierarchical and complex designs, identification of the appropriate level for tests and full reporting of outcomes
☒	☐	Estimates of effect sizes (e.g. Cohen's *d*, Pearson's *r*), indicating how they were calculated

Our web collection on statistics for biologists contains articles on many of the points above.

Software and code

Policy information about availability of computer code

Data collection	Single-cell RNA-seq raw data were processed by the open source Cell Ranger pipeline, version 2.1.1
Data analysis	Single-cell RNA-seq data were analyzed using the open source Seurat (v3.0.0.9) and MAST (v1.6.1) R packages. Parameters are specified in the manuscript.

For manuscripts utilizing custom algorithms or software that are central to the research but not yet described in published literature, software must be made available to editors and reviewers. We strongly encourage code deposition in a community repository (e.g. GitHub). See the Nature Research guidelines for submitting code & software for further information.

Data

Policy information about availability of data

All manuscripts must include a data availability statement. This statement should provide the following information, where applicable:

- Accession codes, unique identifiers, or web links for publicly available datasets
- A list of figures that have associated raw data
- A description of any restrictions on data availability

All data are available from the corresponding author upon request and the data related to the RNAseq experiment are deposited in GEO reference GSE159161.

Field-specific reporting

Please select the one below that is the best fit for your research. If you are not sure, read the appropriate sections before making your selection.

☒ Life sciences ☐ Behavioural & social sciences ☐ Ecological, evolutionary & environmental sciences

For a reference copy of the document with all sections, see nature.com/documents/nr-reporting-summary-flat.pdf

Life sciences study design

All studies must disclose on these points even when the disclosure is negative.

Sample size	Sample size for all in vivo experiments was calculated by the statistics department of Boehringer Ingelheim and verified by the Regierungspräsidium Tübingen (TVV 12-009-G and 14-016-G; 35/9185.81-8)
Data exclusions	no data were excluded
Replication	I confirm that all attempts at replication were successful. See figure legends for details on replicate numbers for specific experiments.
Randomization	animals were randomized into the treatment groups
Blinding	Blinding of this study was not relevant because all samples received numbers and several people were involved in the analysis of the data.

Reporting for specific materials, systems and methods

We require information from authors about some types of materials, experimental systems and methods used in many studies. Here, indicate whether each material, system or method listed is relevant to your study. If you are not sure if a list item applies to your research, read the appropriate section before selecting a response.

Materials & experimental systems

n/a	Involved in the study	
☒	☐	Antibodies
☒	☐	Eukaryotic cell lines
☒	☐	Palaeontology and archaeology
☐	☒	Animals and other organisms
☒	☐	Human research participants
☒	☐	Clinical data
☒	☐	Dual use research of concern

Methods

n/a	Involved in the study	
☒	☐	ChIP-seq
☒	☐	Flow cytometry
☒	☐	MRI-based neuroimaging

Animals and other organisms

Policy information about studies involving animals; ARRIVE guidelines recommended for reporting animal research

Laboratory animals	Female and male wild type C57BL/6J mice and Il1rap-/- mice on the C57BL/6 background or Il-36-/- mice or wild type Balb/c and crl mice, all 8 – 13 weeks of age, were purchased from Charles River (Sulzfeld, Germany or the US). Animals were housed in groups of 5 mice per cage under specific pathogen free conditions in isolated ventilated cages (IVC) at 20 – 25°C and a humidity of 46 – 65% with a dark/night cycle of 12 hours. Mice had free access to water and chow.
Wild animals	the study did not involve wild animals
Field-collected samples	the study did not involve samples from the field
Ethics oversight	All experiments were approved by the animal welfare officers within Boehringer Ingelheim Pharma GmbH and Co KG, as well as by the local authorities for the care and use of experimental animals (Regierungspräsidium Tübingen; TVV 12-009-G and 14-016-G; 35/9185.81-8). Experiments with influenza virus were performed under biosafety level 2 conditions and were in accordance with German national guidelines and legal regulations.

Note that full information on the approval of the study protocol must also be provided in the manuscript.

April 2020
